# GABA signalling modulates plant growth by directly regulating the activity of plant-specific anion transporters

**DOI:** 10.1038/ncomms8879

**Published:** 2015-07-29

**Authors:** Sunita A. Ramesh, Stephen D. Tyerman, Bo Xu, Jayakumar Bose, Satwinder Kaur, Vanessa Conn, Patricia Domingos, Sana Ullah, Stefanie Wege, Sergey Shabala, José A. Feijó, Peter R. Ryan, Matthew Gillham

**Affiliations:** 1 Australian Research Council Centre of Excellence in Plant Energy Biology, Department of Plant Science, Waite Research Institute, School of Agriculture, Food and Wine, University of Adelaide, PMB1, Glen Osmond, South Australia 5064, Australia; 2 School of Land and Food, University of Tasmania, Private Bag 54, Hobart, Tasmania 7001, Australia; 3 Gulbenkian Institute of Science, Oeiras P-2780-156, Portugal; 4 Department of Cell Biology and Molecular Genetics, University of Maryland, College Park, Maryland 20742-5815, USA; 5 CSIRO Plant Industry, GPO Box 1600, Canberra, Australian Capital Territory 2601, Australia

## Abstract

The non-protein amino acid, gamma-aminobutyric acid (GABA) rapidly accumulates in plant tissues in response to biotic and abiotic stress, and regulates plant growth. Until now it was not known whether GABA exerts its effects in plants through the regulation of carbon metabolism or via an unidentified signalling pathway. Here, we demonstrate that anion flux through plant aluminium-activated malate transporter (ALMT) proteins is activated by anions and negatively regulated by GABA. Site-directed mutagenesis of selected amino acids within ALMT proteins abolishes GABA efficacy but does not alter other transport properties. GABA modulation of ALMT activity results in altered root growth and altered root tolerance to alkaline pH, acid pH and aluminium ions. We propose that GABA exerts its multiple physiological effects in plants via ALMT, including the regulation of pollen tube and root growth, and that GABA can finally be considered a legitimate signalling molecule in both the plant and animal kingdoms.

## Abstract (web summary)

GABA is an important neurotransmitter in animals, and while it accumulates in plants under stress, its potential role in plant signalling was poorly understood. Here, Ramesh *et al*. show that GABA rapidly alters the activity of plant ALMT anion channels modifying root growth and stress tolerance.

## Graphical abstract

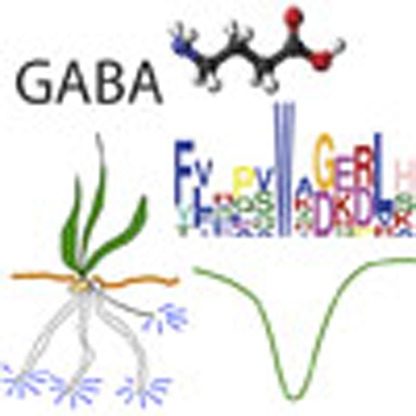

Rapid increases in gamma-aminobutyric acid (GABA) concentration occur in plants in response to extreme temperatures, dehydration, salinity, oxygen stress, mechanical damage, acidosis, virus infection and defence against herbivory^1^,^2^. Elevated GABA concentrations reduce root growth^3^, while GABA gradients are required in the female reproductive tissues to guide pollen tubes to the ovary to ensure successful fertilization^4^. This has led to speculation that GABA signalling occurs in plants, as it does in mammals^1^,^2^,^5^. However, as no molecular components for GABA signalling in plants have been identified^1^,^2^, and there are no plant homologues of mammalian GABA receptors^1^,^2^,^5^, it has remained unclear whether changes in GABA concentration constitute a metabolic response or an adaptive signal^1^,^2^,^6^.

While examining the effect of combining stresses that can modulate plant growth individually^1^,^2^,^3^, we observed an unexpected interplay between acidosis, trivalent aluminium ions (Al^3+^) and GABA accumulation. This finding has led us to the identification of aluminium-activated malate transporters (ALMT) as key transducers of GABA signalling in plants. ALMT form a large multigenic anion channel family exclusive to plants with multiple physiological roles and discrete expression patterns^7^. We demonstrate the impact of GABA regulation of ALMT activity in wheat roots during pH and aluminium stress. More broadly, our findings reveal that GABA-mediated regulation of ALMT proteins underlies a novel signalling pathway that has the potential to translate changes in the concentration of this plant stress metabolite into physiological outputs throughout the plant.

## Results

### GABA regulates malate flux from wheat roots

**Figure 1:**
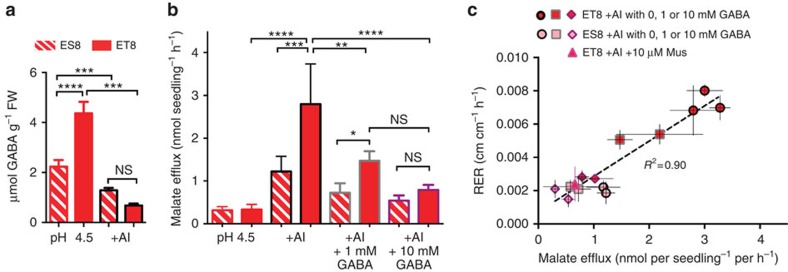
GABA regulates the magnitude of Al^3+^-induced malate flux and the extent of wheat root Al^3+^ tolerance. Hydroponically grown seedlings of near-isogenic wheat lines ET8 (Al^3+^ tolerant) and ES8 (Al^3+^ sensitive)^8^ were used in all experiments, roots were bathed in basal nutrient solution at pH 4.5 ± 100 μM Al^3+^ (+Al) ± 1 or 10 mM GABA, or 10 μM muscimol (Mus) for 22 h. (**a**) The concentration of GABA in ET8 and ES8 wheat roots is decreased in response to Al treatment. (**b**) Malate efflux from wheat roots is increased by Al and decreased in response to Al and GABA treatment in ET8, not ES8 wheat. (**c**) Root malate efflux and root relative elongation rate (RER=(log_e_(length at 22 h)−log_e_(length at 0 h))/22 h) is positively correlated in ET8 in the presence of Al. Both parameters are negatively regulated by GABA and Mus, which phenocopies the response of ES8 to Al. *, **, *** and **** indicate significant differences between genotypes at *P*<0.05, 0.01, 0.001 and 0.0001, respectively, using a one-way ANOVA; NS, not significantly different. The significance comparisons between some groups have been omitted for clarity. All data *n*=5 biological replicates, all error bars are ± s.e.m. All experiments were repeated at least three times.

Acidosis is one of the multitude of stresses that increases GABA concentration in plant cells^2^. Trivalent aluminium ions (Al^3+^) are a frequent co-occurring stress in acid soils^8^. Using near-isogenic lines (NILs) of bread wheat (*Triticum aestivum*, *Ta*) that differ in their Al^3+^ tolerance, we found that GABA concentrations under acidic conditions were significantly higher in roots of the Al^3+^-tolerant NIL ET8 compared with those of ES8, the Al^3+^-sensitive NIL. However, simultaneous application of Al^3+^ and pH 4.5 reduced the GABA concentration in both lines to equivalent levels (Fig. 1a). ET8 and ES8 differ in their abundance of the malate-permeable transporter TaALMT1 in the plasma membrane of root apical cells^8^,^9^. In ET8, where TaALMT1 is abundant, Al^3+^ stimulates sufficient malate efflux at the root tip to chelate Al^3+^ in acid soils and prevent Al^3+^-induced cellular damage; this allows root growth to continue and confers Al^3+^ tolerance^8^,^9^. Therefore, we tested whether exogenously applied GABA had a differential effect on these lines and their tolerance to Al^3+^. At low pH, application of GABA to ET8 roots significantly reduced both Al^3+^-activated malate efflux and root growth in the presence of Al^3+^, whereas GABA had no significant effect on these parameters in ES8 (Fig. 1b,c). As such, we could phenocopy the root growth sensitivity of ES8 to Al^3+^ in ET8 by simultaneously applying GABA at pH 4.5 (Fig. 1c). Overall, our data showed a significant correlation between root growth and malate efflux in the presence of Al^3+^ at pH 4.5 (Fig. 1c)^8^,^9^. We also found that muscimol—a potent analogue of GABA and specific agonist of mammalian GABA_A_ receptors^5^—selectively reduced malate efflux and root growth of ET8 (not ES8) (Fig. 1c). This effect of muscimol could be attenuated by bicuculline, a competitive antagonist of GABA binding to mammalian GABA_A_ receptors^5^ (Supplementary Fig. 1).

### GABA regulates activity of TaALMT1

**Figure 2:**
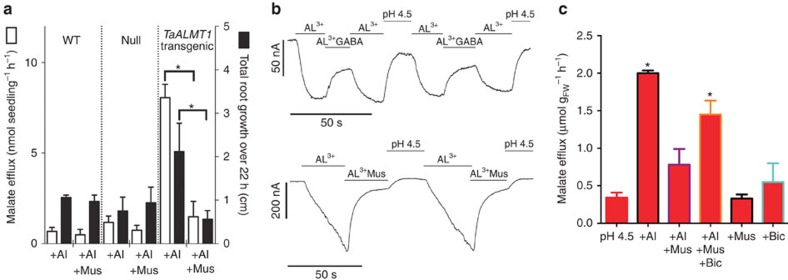
GABA regulates Al^3+^-activated malate efflux through TaALMT1. (**a**) *TaALMT1* expression in barley^10^ increases malate efflux and root growth of barley in the presence of 100 μm Al^3+^ at pH 4.5, over 22 h, but this is negatively regulated by 10 μM muscimol (Mus). (**b**) Representative current traces from *TaALMT1*-injected *X. laevis* oocytes voltage-clamped at −120 mV challenged with 100 μm Al^3+^ ± 100 μm GABA or 10 μM muscimol (Mus) at pH 4.5. (**c**) Malate efflux from *TaALMT1*-expressing BY2 cells^11^ in standard BY2 solution at pH 4.5 ± 100 μm Al^3+^ ± 10 μm Mus ± 100 μm bicuculline (Bic). For controls for **b** and **c**, see Supplementary Fig. 2. *indicates significant differences between genotypes at *P*<0.05 using a two-tailed *t*-test (**a**) or one-way analysis of variance (**c**). Full-TaALMT1 sequence identifier (DQ072260). All data *n*=5 biological replicates (except **b**, which are representative traces from *n*=5). All error bars are ±s.e.m. Transgenic barley experiments were repeated twice, *Xenopus* oocyte experiments were repeated with at least three different frogs and BY2 tobacco cell experiments were repeated thrice.

To test whether muscimol was acting via TaALMT1, we used transgenic barley overexpressing *TaALMT1* (ref. 10). Barley is among the most Al^3+^-sensitive cereals and naturally exhibits a very low level of Al^3+^-stimulated malate efflux compared with Al^3+^-tolerant wheat^10^. However, both Al^3+^-stimulated malate efflux and Al^3+^-tolerance (that is, root growth in the presence of Al^3+^) was increased in barley overexpressing *TaALMT1* when compared with wild-type (WT) barley or null transgenic lines^10^ (Fig. 2a). Both Al^3+^-dependent malate efflux and root growth of barley overexpressing *TaALMT1* was reduced by muscimol, whereas muscimol had no effect on either component in the null lines or WT barley (Fig. 2a). As both muscimol and bicuculline are used as diagnostics for the activity of mammalian GABA_A_ receptors^5^, which are anion channels that are gated by GABA, we examined the effects of these compounds on the anion transport activity of ALMT protein in heterologous expression systems using two-electrode voltage-clamp electrophysiology and flux analysis. Both GABA and muscimol negatively regulated Al^3+^-induced TaALMT1-mediated currents in *Xenopus laevis* oocytes (Fig. 2b; Supplementary Fig. 2a) and TaALMT1-mediated malate efflux in transgenic Tobacco BY2 cells^8^,^11^ (Fig. 2c). Furthermore, bicuculline attenuated the effect of muscimol on Al^3+^-induced fluxes in BY2 cells (Fig. 2c; Supplementary Fig. 2c). We examined the possibility that endocytosis of TaALMT1 contributed to the reduction in malate efflux following GABA treatment, but found no effect of the endocytosis inhibitor Brefeldin A^12^ on Al^3+^-activated malate efflux or the inhibition by muscimol (Supplementary Fig. 3).

**Figure 3:**
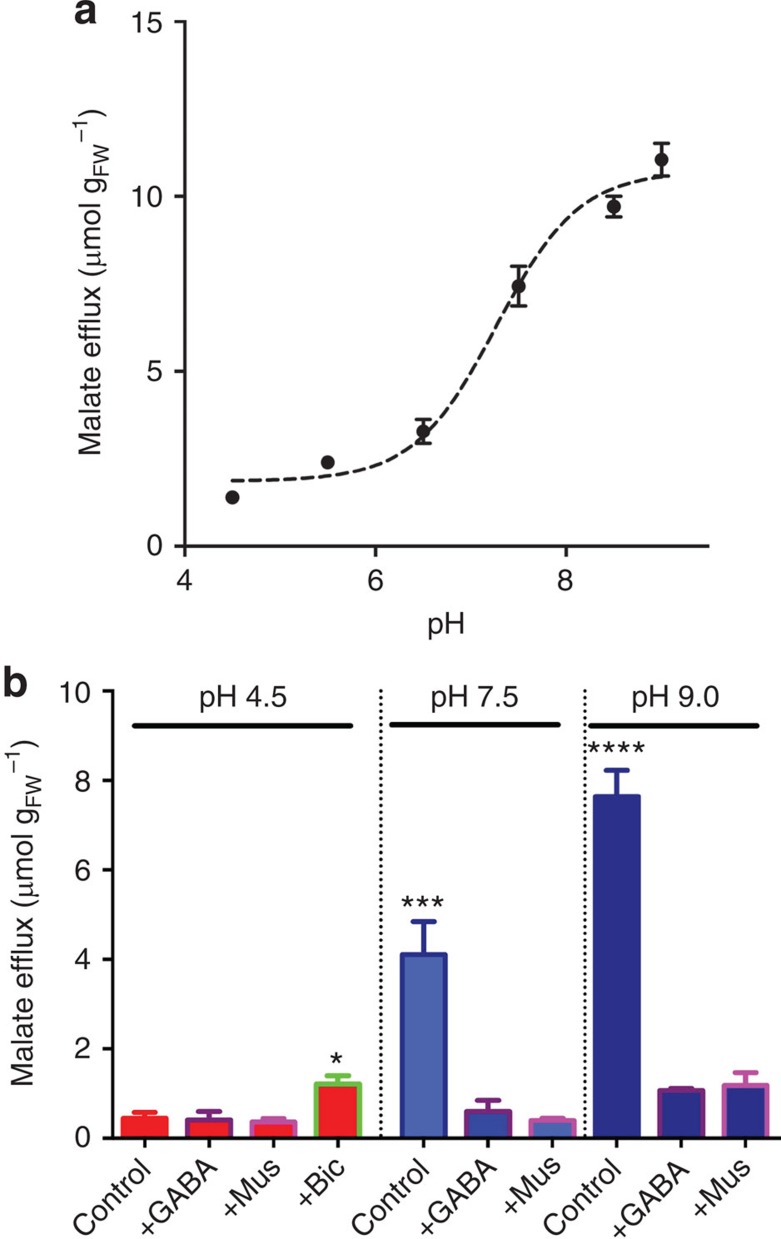
TaALMT1-mediated fluxes are activated by external anions at alkaline pH and regulated by GABA, muscimol and bicuculline. (**a**) Malate efflux from *TaALMT1*-expressing BY2 cells^11^ in standard BY2 solution +10 mM SO_4_^2−^ increases with increasing pH over 22 h. (**b**) Malate efflux from *TaALMT1*-expressing BY2 cells^11^ in the absence of Al^3+^ + 10 mM SO_4_^2−^ is negatively regulated by 100 μm GABA or 10 μm muscimol (Mus) at alkaline pH, and increased by 100 μm bicuculline (Bic) at pH 4.5. *, *** and **** indicate significant differences between genotypes at *P*<0.05, 0.001 and 0.0001, respectively, using a one-way analysis of variance (**b**). All data *n*=3 biological replicates, all error bars are ±s.e.m. All experiments were repeated three times.

**Figure 4:**
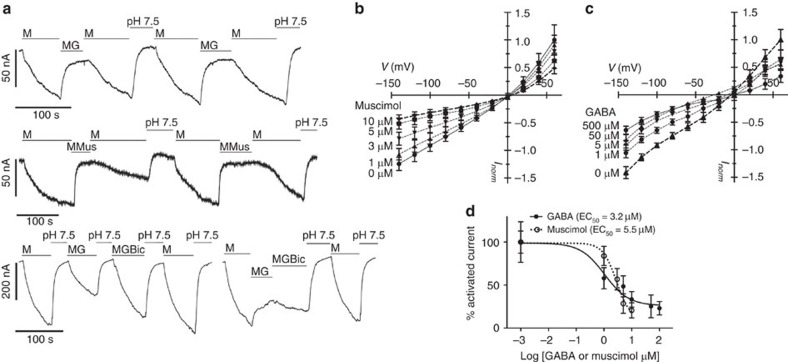
TaALMT1 currents are activated by external malate at pH 7.5 and are regulated by GABA, muscimol and bicuculline. All results from *X. laevis* oocytes injected with *TaALMT1* cRNA bathed at pH 7.5 and measured using two-electrode voltage-clamp electrophysiology. (**a**) Representative current traces at −120 mV; M=10 mM malate; Mus=10 μm muscimol; G=100 μm GABA; Bic=100 μm bicuculline from *n*=5 biological replicates for top and middle traces, *n*=3 for the bottom trace. Response of water-injected control oocytes are shown in Supplementary Fig. 6. (**b**,**c**) Current–voltage relationship of malate-activated current as regulated by muscimol and GABA as indicated applied and recorded 30 s after each solution change. Control-subtracted currents were normalized to the largest mean outward current at +60 mV (*n*=5 independent oocytes for each treatment). (**d**) Concentration dependence of GABA- and muscimol-regulated inward current at −140 mV taken from **b** and **c** (*n*=5 for **b**, and 9 for **c**). All data are water-control subtracted except in **a**. All error bars are ±s.e.m. Experiments repeated with oocytes from at least two different frogs.

During the study of TaALMT1 activity we were surprised to find that at alkaline pH, in the absence of Al^3+^, TaALMT1 could be activated by malate (Fig. 3) or a variety of other anions (Supplementary Fig. 4). Malate-induced malate flux through TaALMT1 was much greater at alkaline pH in both tobacco BY2 cells^11^ (Fig. 3a) and *X. laevis* oocytes expressing *TaALMT1* (Supplementary Fig. 4a), with a *K*_m_ for malate activation of 1.1 mM (Supplementary Fig. 4c). Anion-activated malate efflux was negatively regulated by GABA and muscimol (Fig. 3b; Fig. 4; Supplementary Fig. 5), and this effect was attenuated by bicuculline (Fig. 3b; Fig. 4a; Supplementary Fig. 6). The affinity for GABA and muscimol regulation at pH 7.5 was in the low micromolar range (half-maximal effective concentration=3.2 μM (GABA) and 5.5 μM (muscimol)) (Fig. 4b–d).

Elevated GABA concentrations in plant tissues at low pH (as shown in Fig. 1a) has been proposed to regulate cytosolic pH through the activity of Ca^2+^/CAM-stimulated glutamate decarboxylase, which consumes protons by converting glutamate to GABA^1^; our observations suggest that GABA is playing an additional role. At low pH, in the absence of Al^3+^, GABA and muscimol had no effect on malate efflux, whereas it was significantly increased by bicuculline (Fig. 3b). This suggests that bicuculline can partially override the inhibition that high concentrations of endogenous GABA may have on TaALMT1 activity and malate efflux at acidic pH in the absence of Al^3+^. As such, bicuculline appears to act as a competitive antagonist to GABA action for TaALMT1, as it does for mammalian GABA_A_ receptors. At alkaline pH, endogenous GABA concentration decreased when anions were added externally (Supplementary Fig. 7); this is a condition where greater malate efflux occurs through TaALMT1 in heterologous expression systems (Fig. 3; Supplementary Figs 4 and 5). At alkaline pH, when significant anion activation of TaALMT1 would ordinarily occur, GABA and muscimol were effective in decreasing malate efflux (Figs 3b and 4). While the mechanism that brings about a decrease in GABA concentration at alkaline pH is unclear, the fact that it is low in such conditions may allow for a greater efflux of malate to occur from the roots when in an alkaline environment.

### GABA regulation of TaALMT1 alters plant membrane potential

**Figure 5:**
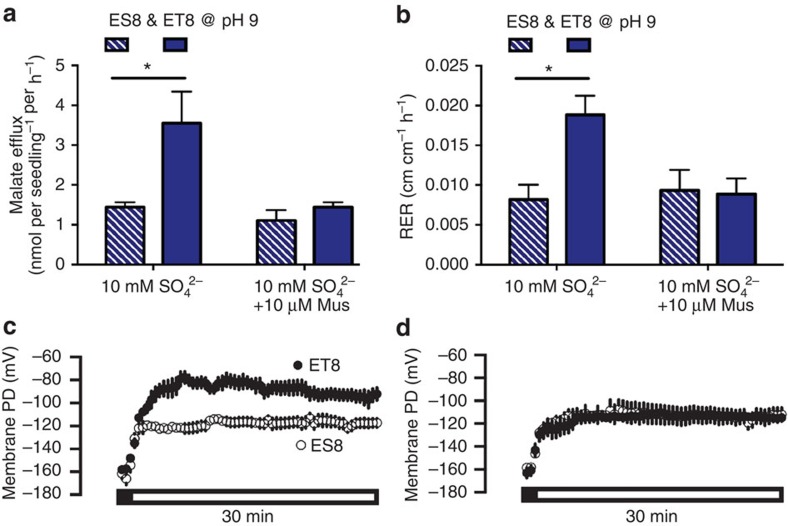
Muscimol-regulated anion-stimulated malate efflux at alkaline pH correlates with wheat root growth and modulates membrane potential. All experiments use ET8 and ES8 seedlings. (**a**) Malate efflux from, or, (**b**) root growth of, wheat roots after 22 h bathed in basal solution at pH 9 + 10 mM SO_4_^2−^ ± 10 μM muscimol (Mus). (**c**) Membrane potential difference (PD) across the plasma membrane of wheat root apical cells in response to 10 mM SO_4_^2–^ at pH 8. (**d**) PD in response to 10 mM SO_4_^2–^ at pH 8 +10 μM muscimol (Mus) treatment. The Black scale bar indicates value prior to treatment and the clear bar is in presence of treatment. * indicates significant differences between genotypes at *P*<0.05 using a one-way a two-tailed *t*-test (**a**,**b**). Biological replicates for **a** and **b** are *n*=5 and **c** and **d** are *n*=4. All error bars are ±s.e.m. Controls are shown in Supplementary Fig. 8. Experiments in **a** and **b** are repeated at least twice.

To test the physiological significance of anion activation of TaALMT1 at alkaline pH, we again used wheat NILs ET8 and ES8. We observed substantial malate efflux from ET8 roots in the presence of an external activating anion, but not from roots of ES8 (Fig. 5a). This alkaline pH-dependent stimulation of malate efflux was inhibited by muscimol (Fig. 5a), and this reduction in malate efflux was again coincident with a reduction of root growth (Fig. 5b). Activation of anion channels will tend to depolarize the plasma membrane potential difference (PD)^13^, so we examined the PD responses of epidermal cells in the root apex of ET8 and ES8 in both alkaline and acid conditions. Under alkaline conditions, addition of external anions depolarized the PD of ET8 to a greater extent than ES8 (Fig. 5c), and concurrent addition of muscimol with anions abolished this difference between lines (Fig. 5d); muscimol application to roots in the absence of channel activation did not significantly affect membrane potential (Supplementary Fig. 8a). The same trends were apparent at pH 4.5 following Al^3+^ treatment—ET8 were more depolarized than ES8, and muscimol abolished the differences between the genotypes (Supplementary Fig. 8b–d). This confirms that wheat roots sense muscimol and anions rapidly and in a TaALMT1-dependent manner, as could be predicted by its effects on TaALMT1 in heterologous expression systems (Figs 2b and 4a). Modulation of PD is known to result in many downstream responses in plants from changes in cell turgor, growth and in gene expression^13^; this suggests that ALMT are prime candidates for transducing GABA signals in plants.

### GABA regulation is a conserved feature of ALMT proteins

**Figure 6:**
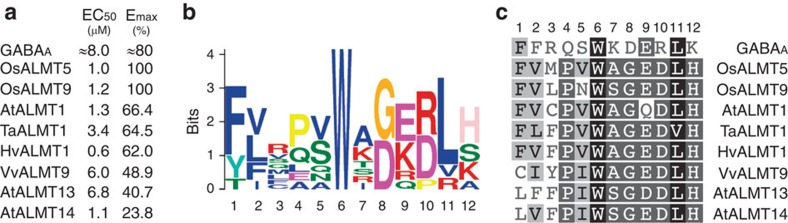
GABA regulation of transport activity is a common ALMT property as is the presence of a putative GABA-binding motif. (**a**) Half-maximal effective concentration (EC_50_) and efficacy (*E*_max_) of GABA regulation of rat GABA_A_ receptors (average) or selected plant ALMT in cRNA-injected *X. laevis* oocytes assayed using two-electrode voltage-clamp electrophysiology (Os=rice; Hv=barley) (full data set, Supplementary Fig. 8). (**b**) Sequence logo of the predicted GABA-binding motif identified using MEME analysis^16^,^18^ (detailed alignment, Supplementary Fig. 9). (**c**) Residues corresponding to logo in proteins from **a** (see Supplementary Figs 10 and 11 for this sequence and residue frequencies at each position within the motif in all other identified ALMT), identical residues shaded (black), 80% similar (grey) and <60% similar (unshaded). Full-sequence identifiers are AtALMT1 (AT1G08430); TaALMT1 (DQ072260); OsALMT5 (Os04g0417000); HvALMT1 (EF424084); AtALMT13 (AT5G46600); AtALMT14 (AT5G46610); OsALMT9 (Os10g0572100); VvALMT9 (XM_002275959). All measurements were carried out at least twice with different frogs.

TaALMT1 was the founding member of the ALMT family^8^, which consists of a large number of anion channel encoding genes present in all plant species—*Arabidopsis* has 14, grapevine 13, soybean 33 and there are 9 in rice. Different family members have been proposed to have specific physiological roles^7^. Despite their name—a legacy of their founding member—many characterized ALMT are not activated by Al^3+^ nor have any role in Al^3+^ tolerance^7^, and they can be activated by millimolar concentrations of anions on the *cis* side of the protein when permeant anions are present on the *trans* side^7^,^13^,^14^,^15^. Some ALMT are preferentially selective for anions other than malate^14^. By examining eight ALMT from five plant species (*Arabidopsis*, wheat, barley, rice and grapevine), our results suggest that GABA regulation of anion-activated currents is a general feature of this family (Fig. 6a; Supplementary Fig. 9a).

### ALMT proteins contain a motif essential for GABA regulation

As regulation of ALMT activity by GABA occurred in the low micromolar range, we attempted to elucidate what residues were important for this effect by comparing sequences of mammalian GABA receptors^5^ and ALMT^7^. GABA is a major inhibitory neurotransmitter, which acts as a signal by regulating ion flow across cell membranes via two classes of receptors, the GABA_A_ and GABA_B_^5^. GABA_A_ receptors consist of multiple subunits that can assemble into a functional homomeric or heteromeric channel^5^. A model of a human α_1_β_2_γ_2_ GABA_A_ receptor was constructed that predicts residues important for binding GABA^16^; many of these residues were subsequently validated as part of a neurotransmitter-binding pocket when the crystal structure of a human homopentimeric β3 subunit GABA_A_ receptor was resolved^17^. Here, using MEME^18^ analysis we discovered a region, 12 amino acids in length, shared between ALMT and the ion channels used to construct the α_1_β_2_γ_2_ GABA_A_ receptor model^16^ (Fig. 6b,c; Supplementary Fig. 10). Sequence analysis suggested that all known ALMT contain this motif and, as is the case for GABA_A_ α- and β-subunits^8^,^16^,^17^ or GABA_A-ρ_ receptors^19^,^20^,^21^, that the aromatic amino-acid residues phenylalanine (F) or tyrosine (Y) contained within the shared motif may be important for GABA efficacy (Fig. 6c; Supplementary Figs 11 and 12). There is debate about the number of transmembrane-spanning domains within ALMT, and their orientation, particularly with regard to the C terminus^7^. However, under current convention the motif spanning amino-acid positions 213–224 in TaALMT1 is predicted to reside near the end of the sixth transmembrane domain, on the external face of the plasma membrane^22^. The rapidity and reversibility of GABA regulation is consistent with GABA interaction occurring from the cell exterior (Figs 2b and 4a).

**Figure 7:**
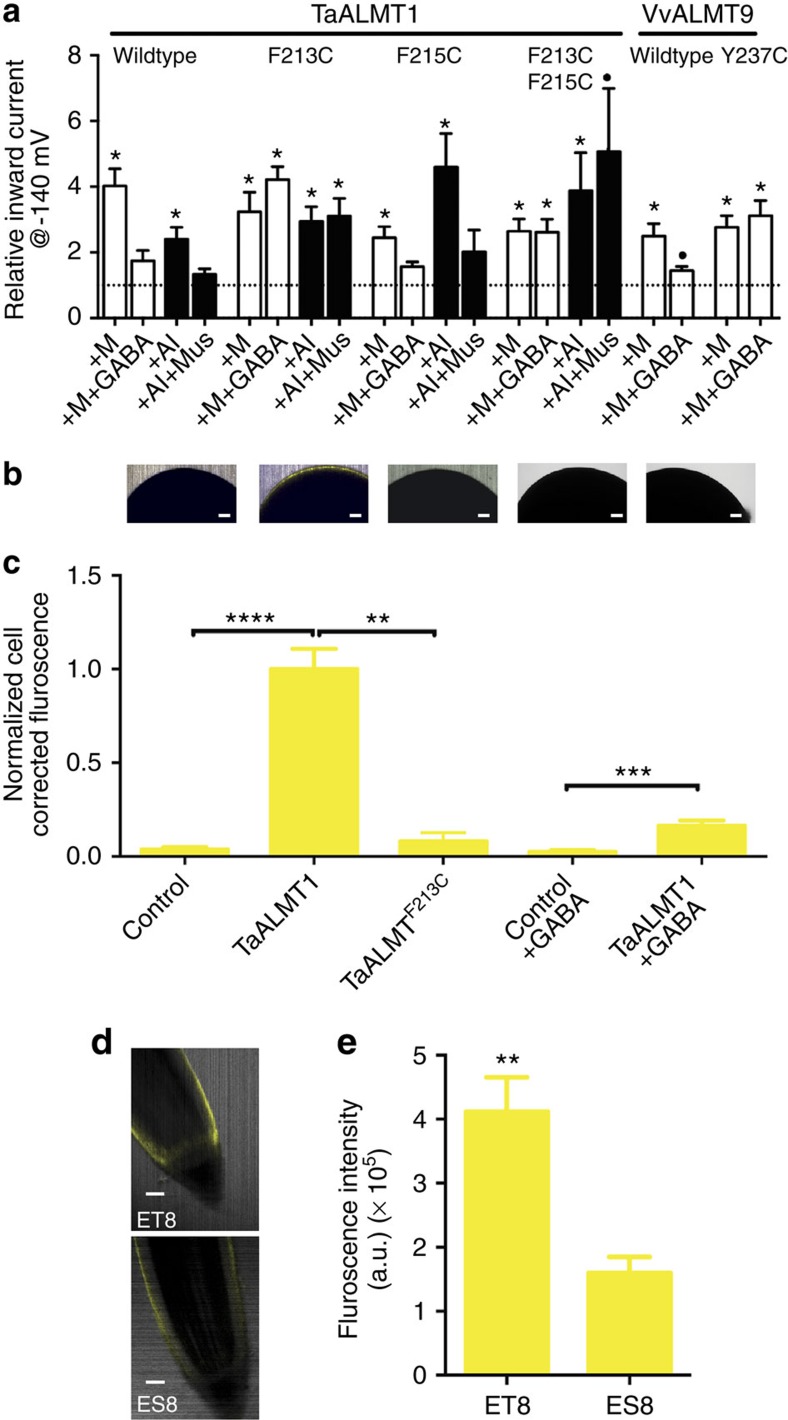
GABA regulation of ALMT transport activity is dependent on an aromatic residue within the predicted GABA-binding motif. (**a**) Sensitivity of wild-type and site-directed ALMT mutants (M, 10 mM malate; GABA, 100 μM), at pH 4.5 (Al, 100 μM; muscimol (Mus), 10 μM) assayed by two-electrode voltage-clamp electrophysiology in cRNA-injected *X. laevis* oocytes. Currents were normalized to −140 mV value in basal solution (at each pH) for each protein (dotted line). For all treatments, *n*=3 for TaALMT1, *n*=4 for TaALMT1^F213C^, *n*=5 for TaALMT1^F215C^, *n*=3 for TaALMT1^F213C,F215C^, *n*=7 for VvALMT9 and *n*=4 for VvALMT9^Y237C^. *indicates significant differences from basal currents within each treatment (P<0.05), ·indicates a significance difference between activated currents (*P*<0.05), using a one-sample *t*-test on log-transformed data. (**b**,**c**) Fluorescence of the plasma membrane of *X. laevis* oocytes after exposure to the muscimol-BODIPY conjugate, control (water injected) (*n*=12), *TaALMT1*- (*n*=14), *TaALMT1*^F213C^-injected (*n*=8) and oocytes co-incubated with GABA and muscimol-BODIPY, control (water injected) (*n*=4) and *TaALMT1*-injected (*n*=6). (**d**,**e**) Fluorescence of wheat roots after exposure to the muscimol-BODIPY conjugate (*n*=5 for each). **, *** and **** indicates significant differences in fluorescence between control, TaALMT1 and TaALMT1^F213C^ at *P*<0.01, 0.001, 0.0001, respectively, using one-way analysis of variance and Tukey's *post hoc* test. All error bars are ±s.e.m., scale bars, 100 μm. Experiments in **a** were carried out at least twice with two different frogs. (**b**,**c**) Measurements were repeated thrice with three different frogs. (**d**,**e**) Fluorescence measurements were carried out twice on roots in different experiments.

To test our predictions about GABA interaction, we performed site-directed mutagenesis on the first and second aromatic residues within this motif in TaALMT1 either in isolation (TaALMT1^F213C^ or TaALMT1^F215C^) or in combination (TaALMT1^F213C/F215C^), and a Y to C conversion of *Vitis vinifera* ALMT9 (VvALMT9^Y237C^), which is the first aromatic residue in that motif. All mutant proteins retained strong activation by external anions (Fig. 7a), however, TaALMT1^F213C^, TaALMT1^F213C/F215C^ and VvALMT9^Y237C^ were not inhibited by 100 μM GABA, whereas inward current through TaALMT1^F215C^ was reduced by GABA (Fig. 7a). The half-maximal effective concentration for GABA regulation for TaALMT1 increased from 3.2 μM to over 1 mM for TaALMT1^F213C/F215C^, and from 5 μM in VvALMT9 to 697 μM in VvALMT9^Y237C^ (Supplementary Fig. 8b). All mutant TaALMT1 also tested positive for Al^3+^ activation, and only TaALMT1^F215C^ retained sensitivity to 10 μM muscimol (Fig. 7a). We observed a strong physical interaction of a fluorescent muscimol conjugate^23^ with the surface of *X. laevis* oocytes when injected with *TaALMT1*, but not following *TaALMT1*^F213C^ or water injection (Fig. 7b,c). ALMT activation by anions or Al^3+^ are processes believed to be dependent upon amino-acid residues identified within the N- and C terminus^24^,^25^; our findings are consistent with the GABA-responsive motif being distinct from these regions. We further investigated the interaction of GABA with ALMT by co-exposing *X. laevis* oocytes injected with *TaALMT1* with the fluorescent muscimol conjugate^23^ and GABA. We observed a significant lower fluorescence signal from *TaALMT1*-injected oocytes co-incubated with the fluorescent muscimol conjugate and GABA compared with those not exposed to GABA; however, the fluorescence was significantly greater from co-incubated oocytes than from water-injected control oocytes (Fig. 7b,c). These findings suggest that GABA and the fluorescent muscimol conjugate are in direct competition for their association with TaALMT1. The evidence that muscimol and GABA directly associate with TaALMT1 is also strengthened by the *in planta* observation that the ET8 wheat root apex fluoresces to a greater extent when exposed to the fluorescent muscimol conjugate compared with the root apex of ES8 (Fig. 7d,e). The magnitude of fluorescence therefore appears to be relative to the quantity of TaALMT1 protein present in each line, with TaALMT1 more abundant in the root apex of ET8 (refs 8, 9).

### ALMT are key transducers of GABA signalling in plants

**Figure 8:**
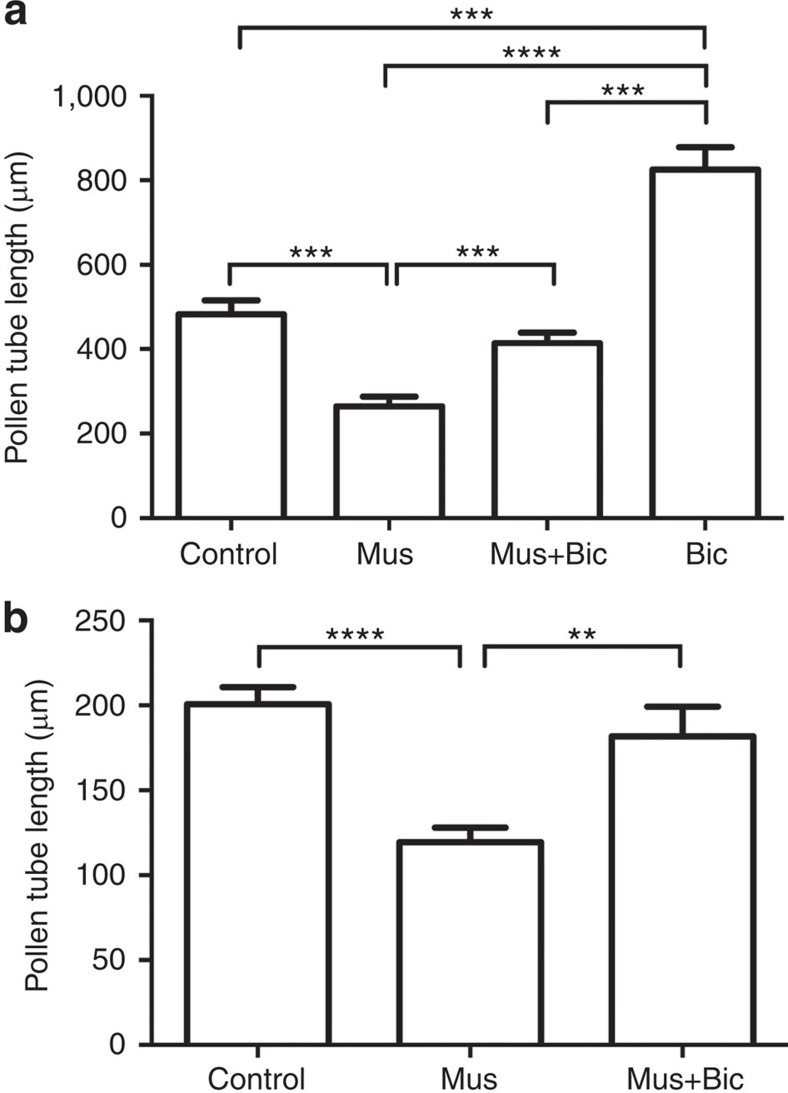
Muscimol reduces *Arabidopsis thaliana* and *Vitis vinifera* pollen tube elongation *in vitro*, and bicuculline antagonizes muscimol regulation. (**a**) *In vitro Arabidopsis* pollen tube elongation after 3 h±treatments (*n*=499). Mus (20 μM muscimol and Bic (200 μM bicuculline). (**b**) *In vitro* grapevine pollen tube elongation after 6 h ± treatments, mean Δ from control mean±s.e.m. (12.5±0.16 mm) (*n*=157–193 per treatment). **, *** and **** indicate significant differences between genotypes at *P*<0.01, 0.001 and 0.0001, respectively, using a one-way analysis of variance and Tukey's *post hoc* test. All error bars are ±s.e.m. (**b**) The experiments were replicated at least three times.

It was previously proposed that Al^3+^ activation constitutes a specialized diversification of the ALMT family^7^; the majority of ALMT are instead suggested to underlie voltage-dependent quickly-activating anion channel/rapidly activating (R-type) channel activity across the plasma membrane and tonoplast of most plant cell types^7^,^13^,^14^. R-type channels are commonly associated with cell signalling in plants in multiple cell types^13^,^26^. For instance, GABA gradients are required in female reproductive tissues to guide pollen tubes to the ovary to ensure successful fertilization^4^. Here, we provide evidence that GABA-regulated growth of pollen tubes^4^ is mediated through GABA-gated ALMT, as muscimol reduced pollen tube growth and this growth reduction was attenuated by bicuculline (Fig. 8).

## Discussion

Our findings in wheat roots suggest that GABA negatively regulates the activity of TaALMT1—a protein that is constitutively present in root apical cells^8^,^9^—to prevent malate efflux from roots under certain conditions. This occurs under acidic conditions (for example, Fig. 1), and is likely to occur under a range of other stresses that increase GABA concentration in plant tissues such as cold, salt or heat^1^,^2^. Excessive carbon efflux would be a disadvantage when conservation of valuable plant reserves of reduced carbon and energy was required, as this would be essential for continued growth and stress tolerance. We also discovered that malate was excreted from wheat roots through TaALMT1 at alkaline pH, with greater malate efflux linked to greater root growth. While *TaALMT1* is commonly associated with aluminium tolerance in acid soils, it was recently found that bread wheat genotypes with *TaALMT1* also have higher yields in alkaline soils^27^,^28^. The excretion of malate at high pH, probably coupled to the efflux of protons would tend to buffer the cell wall space to a lower and more conducive pH for the cell wall loosening that is required for root extension, and for nutrient uptake. Therefore, malic acid efflux would be advantageous in alkaline soils. The observation that GABA tightly regulates root ALMT activity therefore has implications for how plants regulate C sequestration into the rhizosphere, a major energy source for the soil microbiome, and how this might be affected by stress.

GABA and the enzymes that regulate the GABA shunt pathway play a key role in primary C/N metabolism by modulating the flux of carbon and energy through the TCA cycle^29^. The discovery of anion channels in plants gated by physiologically relevant GABA concentrations^2^ links plant metabolism with signalling under both stressed and non-stressed conditions. This is a conceptual advance that opens novel research avenues for crop improvement, particularly for altering stress tolerance, as GABA increases rapidly during multiple stresses^2^. ALMT form a multigenic protein family with different members having diverse expression throughout plant tissues. The concentrations of anions required to activate ALMT are commonly encountered within plant tissue, suggesting that ALMT are ordinarily active in cells or are at least primed for activation. This is consistent with the many physiological roles that are emerging for ALMT encompassing stress tolerance, mineral nutrition, vacuolar malate accumulation and stomatal aperture control^8^,^13^,^14^,^15^. As ALMT activity, or the inhibition of their activity, can directly affect membrane potential with downstream physiological responses^13^ (Fig. 5c,d), it is likely that the different family members more broadly transduce GABA effects throughout plant tissues.

Our results invoke interesting questions regarding the evolution of amino-acid signalling across kingdoms. GABA exerts its inhibitory effect in mature brain neurones by activation of Cl^–^ currents through GABA_A_ receptor channels. This tends to hyperpolarize the membrane potential and inhibits excitability. In plants the anion equilibrium potential is normally very positive so that when ALMT proteins are activated there is a depolarization (that is, Fig. 5c). Plant action potentials are largely based on activation of voltage-dependent anion channels^30^. Thus GABA inhibition of ALMT will tend to hyperpolarize the membrane potential and decrease excitability, similar to the effect of GABA in animal neurones. However, despite having a similar effect on membrane potential, the proteins that transduce GABA signals in plants and animals are not orthologues. They contain no extensive regions of sequence homology, but contain a small region that shares significant similarity to a region that has been previously associated with GABA interaction in mammalian GABA_A_ receptors^16^,^17^ (Fig. 6). The association of muscimol-BODIPY fluorescence with membranes that contain a significant GABA-responsive ALMT protein *in planta* and heterologous systems, and not with membranes containing a site-directed ALMT mutant with diminished GABA sensitivity (Fig. 7), suggests that GABA may interact directly with this similar motif in ALMT and GABA_A_ receptors. However, like in GABA_A_ receptors it is highly likely that other parts of the protein, either of the monomer or between interacting monomers in a multimeric form, constitute the binding region. Interacting regions with the region we have identified will be the subject of future research. It is unclear whether the existence of a similar GABA-binding motif in multiple kingdoms is an example of convergent evolution or the recruitment of an ancestral GABA motif into distinct proteins. Exploration of these possibilities would form an interesting area of future study, as would the employment of methods with increased sensitivity for assessing the variation in GABA-binding affinity among ALMT using direct binding assays or the substituted-cysteine accessibility method^19^.

It is also interesting to note that compounds derived from plants (bicuculline) or mycorrhizal fungi (musicmol), that are known to regulate mammalian GABA receptors, can affect ion transport in plants, and as a consequence plant growth. In light of this, it would seem sensible to reassess the roles of these and similar compounds in nature. Furthermore, since plants, animals and fungi respond to GABA, it has been suggested that GABA can facilitate communication between kingdoms^31^. Our data endorse ALMT as prime candidates to mediate GABA-based signalling in plants.

## Methods

### Chemicals

All chemicals were supplied by Sigma, except muscimol and muscimol-BODIPY TMR-X conjugate^23^ supplied by Life Technologies. Bicuculline is unstable at alkaline pH^32^, so its effects were tested over long time courses only at acidic pH (>1 h), not under alkaline conditions.

### Preparation of ALMT and site-directed mutants

*ALMT* were cloned and mutagenized using primers listed in Supplementary Table 1.The coding nucleotide sequence for various ALMT (AB081803.1 (TaALMT1); EF424084 (HvALMT1); AL606598 (OsALMT5); AAL86482 (OsALMT9); NM_124030 (AtALMT13); NM_124031 (AtALMT14); XM_002275959.1 (VvALMT9)) were cloned using primers listed in Supplementary Table 1 from complementary DNA made from RNA extracted from the target plant into pGEMHE-DEST (GATEWAY enabled)^33^. *TaALMT1* and *VvALMT9* in pGEMHE-DEST were used as a template for mutagenesis. Primers listed in Supplementary Table 1 were designed based on the QuikChange Site Directed Mutagenesis Kit PCR protocol (Stratagene). The amplified mutagenized products were transformed into *Escherichia coli* and confirmed through sequencing. Plasmid DNA was extracted using the Mini Prep kit from Sigma, and 1 μg of plasmid DNA was linearized with the restriction enzyme Nhe1 except for *VvALMT9*, which was linearized using Sph1. Capped complementary RNA (cRNA) was synthesized using the mMESSAGE Mmachine T7 Kit (Ambion) as per the manufacturer's instructions.

### Voltage-clamp electrophysiology and confocal microscopy

Electrophysiology was performed on *X. laevis* oocytes 2 days post injection with water/cRNA^24^,^33^. Oocytes were injected with 46 nl of RNase-free water using a micro-injector (Nanoject II, automatic nanolitre injector, Drummond Scientific) ± 16–32 ng cRNA. Sodium malate (10 mM, pH 7.5) was injected into oocytes 1 h before measurement. Basal external solutions for anion activation contained 0.7 mM CaCl_2_ and mannitol to 220 mOsm kg^−1^, ± 10 mM malic acid and other treatments outlined in the figure legends, buffered with 5 mM BTP/MES from pH 4.5 to 9.0. Aluminium activation was carried out in ND88 (ref. 15). All data are subtracted from mean currents from water-injected controls, except where stated. For confocal imaging, *X. laevis* oocytes or wheat roots were incubated in basal external solution (pH 7.5) with the addition of 10 μM muscimol-BODIPY TMR-X conjugate^23^ for 10 min, 2 days post injection, then washed in basal solution for 7 min and visualized under a Zeiss Confocal microscope with Pascal LSM 5 software with excitation at 514 nm and emission at >530 nm. The images were analysed with LSM 5 image examiner (Zeiss). Fluorescence was quantified using the ImageJ software (NIH)^34^. In all *X. laevis* oocyte experiments, solutions were applied to gene-injected oocytes in the same order as controls (water injected). Randomly selected oocytes were alternated between control and gene injected to limit any bias caused by time-dependent changes after gene injection or malate injection. The University of Adelaide Animal Ethics Committee approved the *Xenopus laevis* oocyte experiments; project number S-2009-044B.

### Root assays

NILs of wheat ET8 and ES8 (ref. 8), and barley^10^ were surface sterilized, and 4-day-old seedlings were placed in a microcentrifuge tube with roots immersed for 22 h in 3 mM CaCl_2_, 5 mM MES/BTP to pH 4.5–9.0 ± treatments, with total root length and number measured at 0 and 22 h. For root flux assays and growth measurement, experiments were carried out wherein the identity of the treatment solutions was unknown to the person performing the experiments to remove any bias. Malate concentrations were measured on an OMEGA plate-reading spectrophotometer (BMG) following the K-LMALR/K-LMALL assay^11^ (Megazyme). One hundred microlitre of the treatment solution collected from roots, or after centrifugation of BY2 samples at 500g in a desktop microcentrifuge, was added to a mastermix containing the various components of the K-LMALR/K-LMALL assay^11^ (Megazyme) kit as per the manufacturer's instructions. The change in absorbance at 340 nm was used to calculate the concentration of malate in the samples. GABA concentrations were measured, also on the OMEGA plate-reading spectrophotometer, following the GABase enzyme assay^35^. Briefly, 5 mm of root tips were excised and snap frozen in liquid nitrogen after seedlings were subjected to treatment solutions for 22 h. The root tips were ground in liquid nitrogen and known weight was added to methanol and incubated at 25 °C for 10 min. The samples were vacuum dried, resuspended in 70 mM LaCl_3_, pelleted at 500g in a desktop microcentrifuge and precipitated with 1 M KOH. These samples were recentrifuged at 500g and 90.34 μl of supernatant was assayed for GABA concentrations using the GABase enzyme from Sigma as per the manufacturer's instructions. Membrane potential measurements were carried out with seedlings placed horizontally in a custom chamber^36^. Plants were allowed to stabilize in the above solution (without treatments) for 60 min prior to measurement. Measurements were made 1 mm from the meristem in the elongation zone. Fine-tipped borosilicate glass microelectrodes (Clark Electromedical Instruments) were filled with 1 M KCl and connected to a Microelectrode Ion Flux Estimation (MIFE) amplifier^36^ via a Ag–AgCl half-cell and inserted into the root tissue with a manually operated micromanipulator (Narishige). Voltage recordings were made on the MIFE CHART software^36^.

### Tobacco BY2 malate efflux

Tobacco suspension cells (*Nicotiana tabacum* L. cv. Samsun, a cell line SL) transformed with the *TaALMT1* gene from wheat (cell line 4) or an empty vector (cell line 9), originally generated by Takayuki Sasaki at Okayama University^11^, were grown in Murashige and Skoog's medium on a rotary shaker (∼100 r.p.m.) until the logarithmic phase. Aliquots of suspension cells containing ∼1 g of cells were centrifuged and gently resuspended in a basal BY2 solution^11^. *TaALMT1*-expressing or vector-control tobacco-BY2 suspension cells (0.15 g) were placed in 3 ml of 3 mM CaCl_2_, 3 mM sucrose and 5 mM MES/BTP to pH 4.5–9.0 ± treatments in 50 ml tubes on a rotary shaker for 22 h, unless otherwise stated. Malate fluxes were measured as stated above.

### Pollen tube experiments

Pollen tube assays were followed^4^ with modification. Pollen was harvested from *Vitis Vinifera* cv. Shiraz (clone BVRC17) grapevine cuttings^37^ with 50 flowers (10 flowers from 5 plants) harvested on the day of anthesis and fixed in liquid N_2_ for storage at −80 °C before use. Pollen was transferred using a fine brush onto cavity slides containing 1 ml of pollen in modified germination medium^38^ containing 15% sucrose, 1.27 mM Ca(NO_3_)_2_.4H_2_O, 1 mM KNO_3_, 0.81 mM MgSO_4_.7H_2_O and 1.6 mM H_3_BO_3_, made in 1 mM MES and buffered to pH 5.6 using 1 M TRIS) and allowed to germinate at 25 °C within 1 h of thawing. The slides were placed in Petri plates containing moistened tissue paper and sealed with parafilm to ensure humidity in the Petri plates. *Arabidopsis thaliana* ecotype Col-0 were grown in hydroponically^39^. Pollen grains from open flowers WT Col-0 were suspended on modified *Arabidopsis* pollen germination medium^40^ containing the basic components (0.01% boric acid, 5 mM CaCl_2_, 5 mM KCl, 1 mM MgSO_4_, 250 μM HEPES, 10% sucrose and pH 7.5–7.8), and incubated at 22 °C. Pollen germination medium (final volume 20 ml) was always prepared fresh from 100 × stock solutions of the main components using autoclaved MilliQ water (Millipore). Multiple representative images of the entire pollen population were taken under a ZEISS Axiophot microscope with a TOUPCAM UCMOS05100KPA camera and ToupView software (ToupTek). All pollen tube lengths were quantified with ImageJ software (NIH).

### Statistics

All graphs and statistics were performed in Graphpad Prism 6. All data shown are mean±s.e.m. Asterisks indicate significance between values as determined by one-way analysis of variance with Tukey's *post hoc* test, unless otherwise stated.

## Additional information

**How to cite this article:** Ramesh, S. A. *et al*. GABA signalling modulates plant growth by directly regulating the activity of plant-specific anion transporters. *Nat. Commun.* 6:7879 doi: 10.1038/ncomms8879 (2015).

## Supplementary Material

Supplementary Information Supplementary Figures 1-12 and Supplementary Table 1
