# Corrigendum: GABA signalling modulates plant growth by directly regulating the activity of plant-specific anion transporters

**DOI:** 10.1038/ncomms9293

**Published:** 2015-08-28

**Authors:** Sunita A. Ramesh, Stephen D. Tyerman, Bo Xu, Jayakumar Bose, Satwinder Kaur, Vanessa Conn, Patricia Domingos, Sana Ullah, Stefanie Wege, Sergey Shabala, José A. Feijó, Peter R. Ryan, Matthew Gilliham

*Nature Communications* 6, Article number: 7879 10.1038/ncomms8879 (2015); Published: 07292015; Updated: 08282013.

The original version of this Article contained a typographical error in the spelling of the author Matthew Gilliham, which was incorrectly given as Matthew Gillham. This has now been corrected in both the PDF and HTML versions of the Article.

