# Supplementary material for: GABA signalling modulates plant growth by directly regulating the activity of plant-specific anion transporters
Source: Nat Commun. 2015 Jul 29;6:7879. doi: 10.1038/ncomms8879 (PMC4532832; doi:10.1038/ncomms8879)
Supplement: Supplementary Information — Supplementary Figures 1-12 and Supplementary Table 1 [file ncomms8879-s1.pdf]

# Supplementary Data

## Supplementary Figures

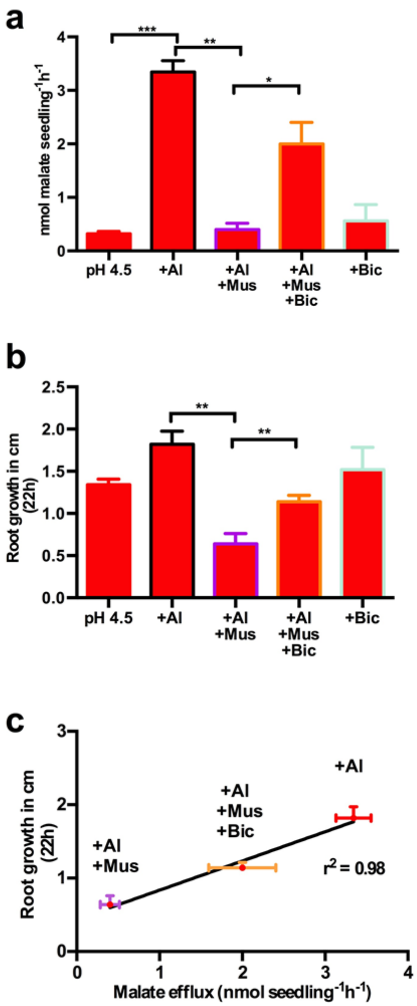


**Supplementary Figure 1 Bicuculline abolishes muscimol regulation of $Al^{3+}$ tolerance of ET8 wheat roots[8].** Muscimol (Mus, 10 µM) in the presence of 100 µM $AlCl_3$ (Al) at pH 4.5 inhibited **a**, malate efflux and **b**, growth, whereas 100 µM bicuculline (Bic) abolished the effect of muscimol. **c**, linear regression showing strong significant correlation between malate flux and growth in presence of combinations of Al, Mus and Bic. All data n = 5, *, ** and *** indicates significance at 0.05, 0.01 and 0.001 respectively of relevant comparisons using a two-tailed t-test.

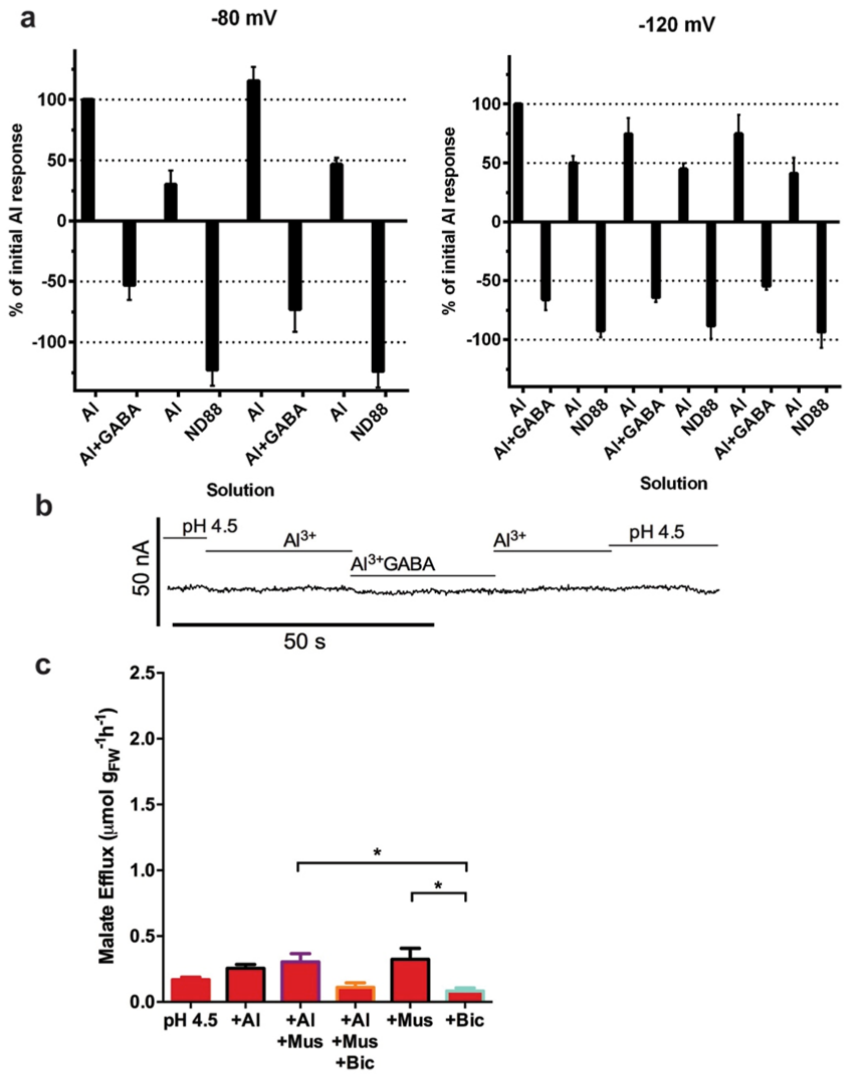


**Supplementary Figure 2 GABA and muscimol regulate $Al^{3+}$-activated malate currents and fluxes through TaALMT1. a,** Percentage activation of malate efflux by $Al^{3+}$ and inhibition of this current by 100 µM GABA in *TaALMT1*-injected *X. laevis* oocytes. Sequential solution changes (30 s in each solution) were made over 6 min at -80 mV (left) and -120 mV (right). These experiments demonstrate reversibility and lack of rundown. n = 8 independent oocytes. **b**, Representative current traces at -120 mV of water-injected control *X. laevis* oocytes bathed in ND88 at pH 4.5 ± 100 µM $Al^{3+}$ ± 100 µM GABA, control for Fig. 2b. **c**, Malate fluxes from vector control transformed BY2 cells, control for Fig. 2c, n=5. Scaling equivalent to Fig. 2c.

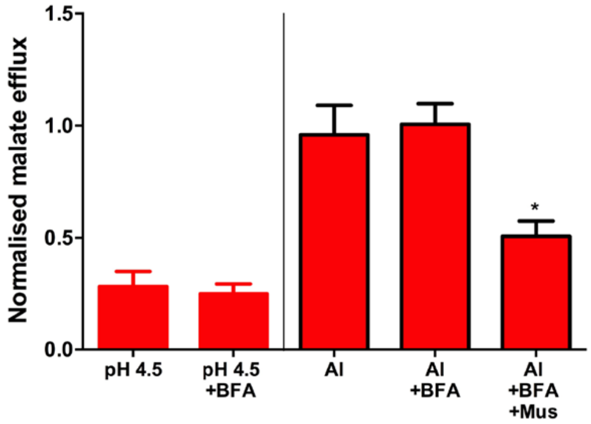


**Supplementary Figure 3 The endocytosis inhibitor Brefeldin A (BFA) had no effect on the $Al^{3+}$-activated malate efflux carried by TaALMT1 or its muscimol inhibition.** Tobacco BY2 cells expressing *TaALMT1* were placed in 3 mM $CaCl_2$ + 5 mM MES, pH 4.5 overnight for 20 h followed by transfer to same solution but ± BFA (100 µM) or $AlCl_3$ (100 µM) ± BFA ± Musc (10 µM) for 1 h. Solutions were buffered with BTP. Supernatant was harvested and assayed for malate fluxes. All fluxes were normalized to value for +Al.

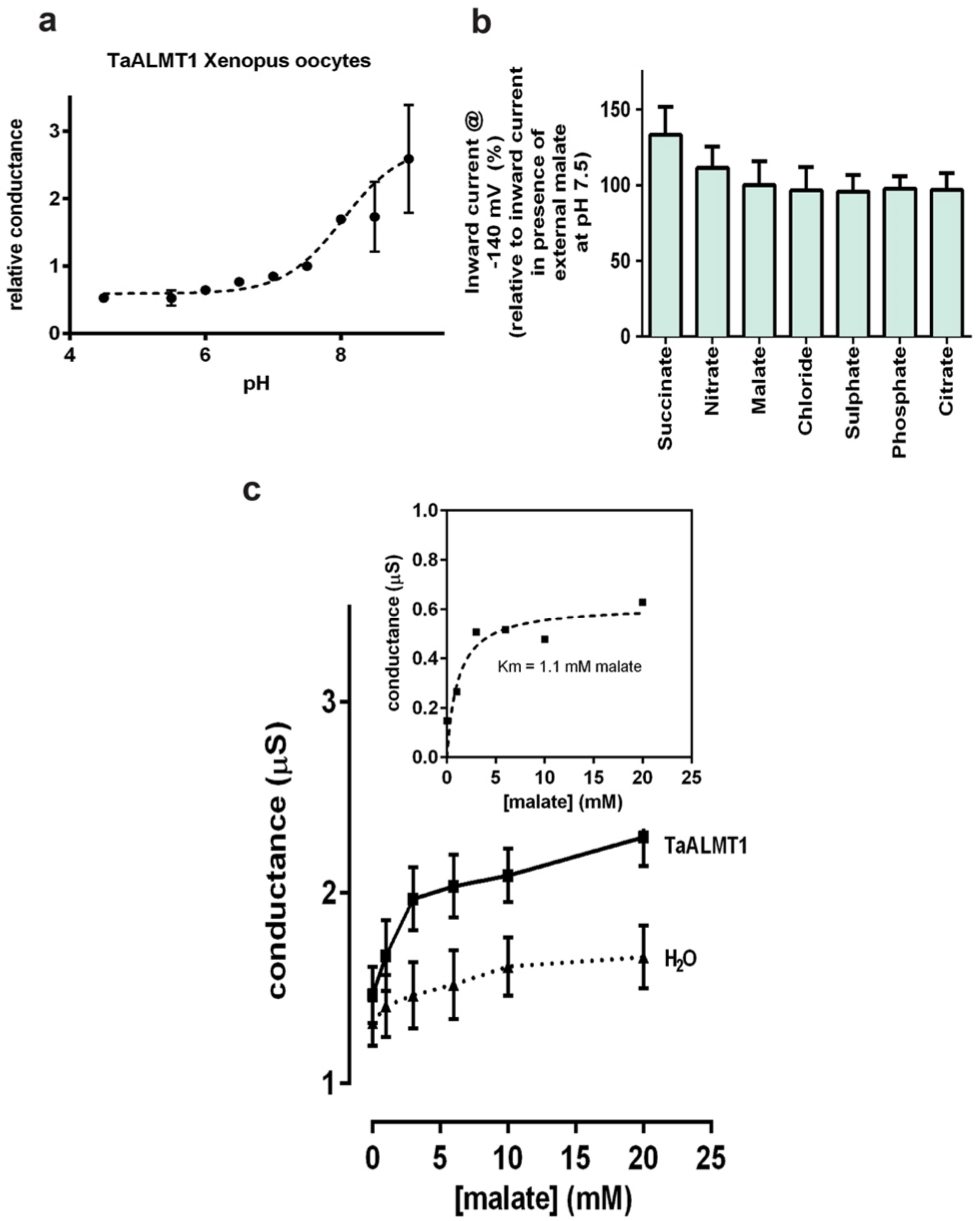


**Supplementary Figure 4 External anions activate TaALMT1 in *X. laevis* oocytes**. **a,** Relative slope conductance of TaALMT1-injected oocytes, normalised to value at pH 7.5 (in the absence of $Al^{3+}$), n = 5. **b**, No significant difference between activation of TaALMT1 by 10 mM of each anion applied externally using a one-way ANOVA. **c,** Slope conductance (–100 to –140 mV) vs current of *TaALMT1*- or water-injected *X. laevis* oocytes. Michaelis-Menten fit of control subtracted TaALMT1-mediated currents (inset). All oocytes were pre-injected to contain ~10 mM malate.

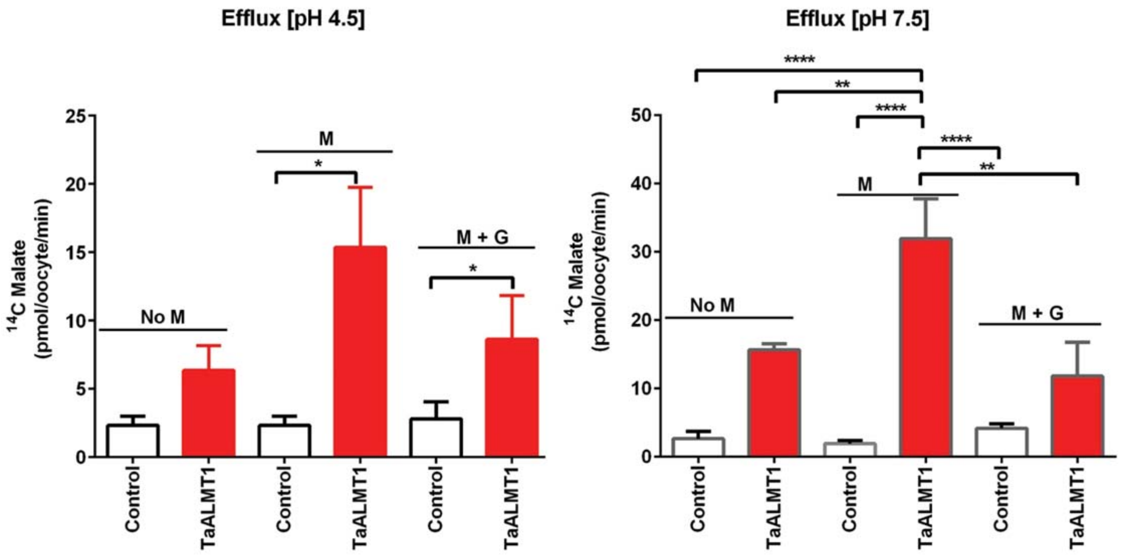


**Supplementary Figure 5 Anion activation of TaALMT1 is greater at alkaline pH and modulated by GABA.** *X. laevis* oocytes injected with TaALMT1 cRNA or water were preloaded with [$^{14}$C] malate and exposed to solution containing malate (M, 10 mM) ± GABA (G, 100 μM). Malate efflux was measured at pH 7.5 or 4.5. All data is mean efflux of radioactive malate from the oocytes 2 min after injection (n = 5). *, **, and **** indicates significant differences between genotypes at p<0.05, 0.01, and 0.0001 respectively using a one way ANOVA and tukey's post-hoc test.

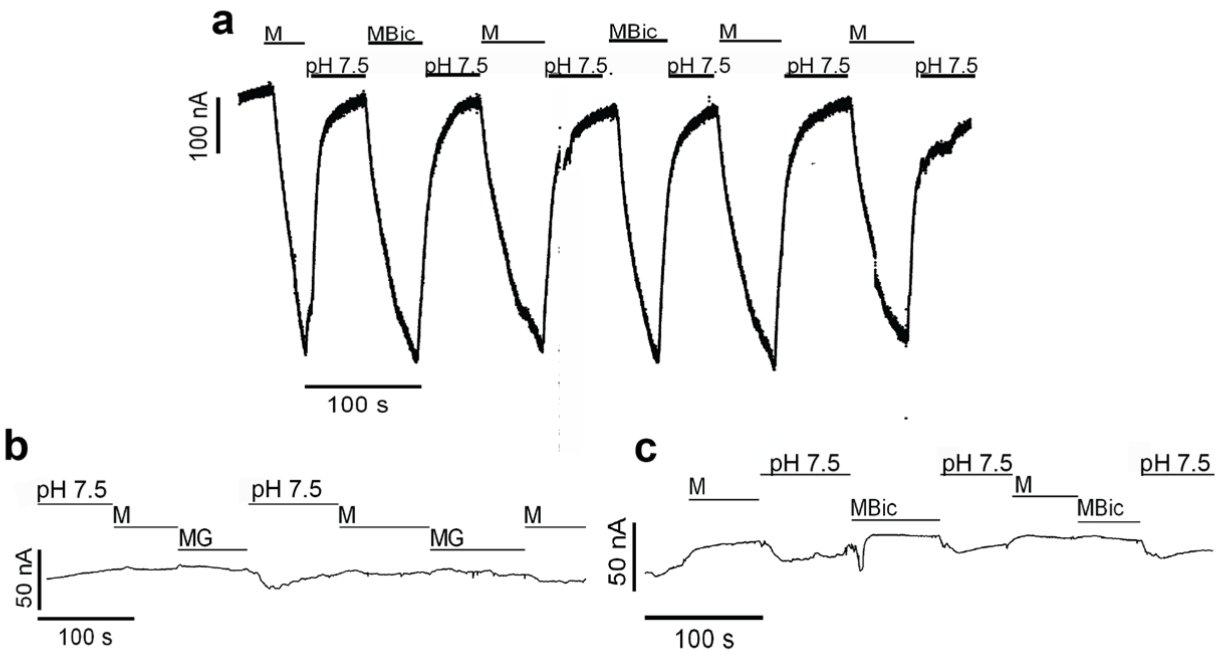


**Supplementary Figure 6 GABA negatively regulates anion-activated malate currents through TaALMT1.** In *TaALMT1*-injected *X. laevis* oocytes representative current traces at -120 mV showing **a,** 100 μM bicuculline (Bic) has no effect on malate activated currents in the absence of GABA at pH 7.5. **b**,**c**, In water injected control oocytes, malate, bicuculline and GABA have no effect on currents at -120 mV. These traces are the controls for Fig. 4a.

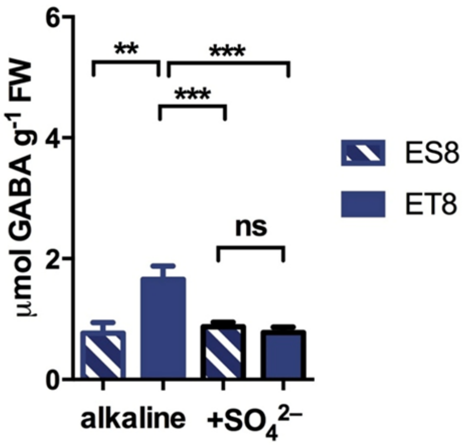


**Supplementary Figure 7 Effect of alkaline pH on GABA concentration in wheat NIL ET8 and ES8[8].** GABA concentration of wheat roots bathed in basal solution at pH 7.5 ± 10 mM $SO_4^{2-}$. n = 11 from 3 individual experiments. ***, and **** indicates significant differences between genotypes at p< 0.001 and 0.0001 respectively using a one way ANOVA and tukey's post-hoc test, ns, not significant. Scaling equivalent to Fig. 1a.

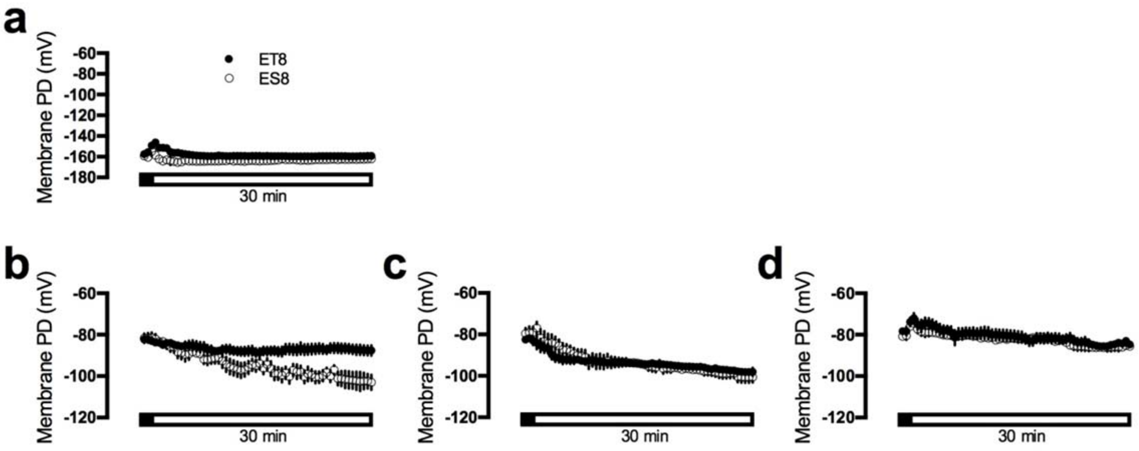


**Supplementary Figure 8 Membrane potential difference (PD) across plasma membrane of wheat NIL ET8 and ES8[8] root apical cells in response to muscimol, and to $Al^{3+}$. a,** Control for Fig. 5c,d; no differential response between genotypes when exposed to 10 μM muscimol in the absence of a transactivating anion, 10 mM $SO_4^{2-}$ at pH 8, n = 3 per genotype. **b**, Membrane potential after 100 μM $Al^{3+}$ treatment addition at pH 4.5. **c,** membrane potential after simultaneous addition of 100 μM $Al^{3+}$ and 10 μM Mus treatment at pH 4.5, n = 7 per genotype. **d**, Membrane potential after 10 μM Mus treatment at pH 4.5, n = 6 per genotype. Black scale bar indicates value prior to treatment, clear bar is in presence of treatment.

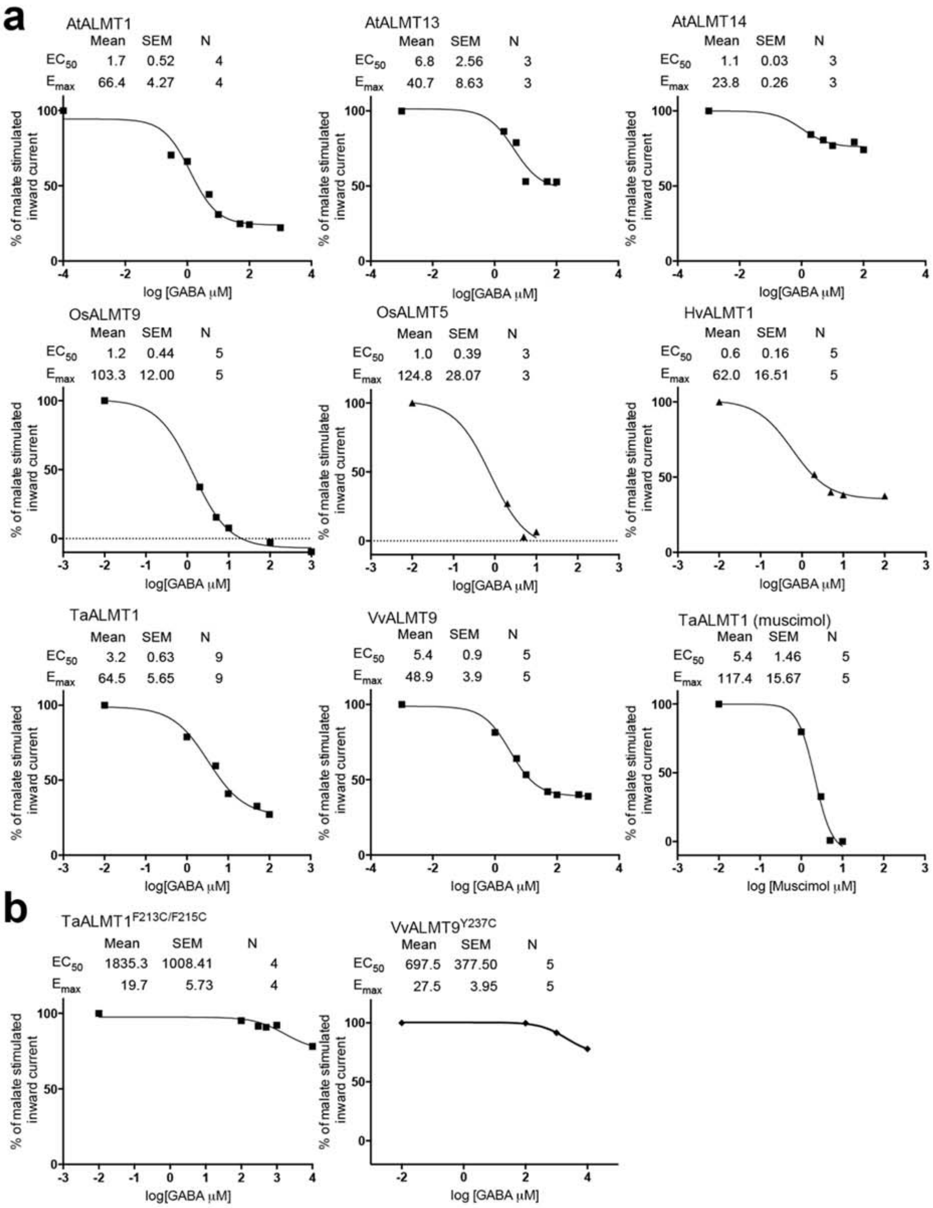


**Supplementary Figure 9. The affinity of GABA (and muscimol) regulation of anion-activated currents through various plant ALMT expressed in *X. laevis* oocytes. a,** wildtype proteins **b,** mutagenised TaALMT$^{F213C/F215C}$ or VvALMT9$^{Y237C}$. All experiments performed in basal solutions + symmetrical 10 mM malate with current at –140 mV taken 30 s after each solution change. Data is control subtracted, presented is example of one oocyte for each gene, and the table shows statistics for repeat experiments

| Name | Start | *p*-value | Sites | | |
|---|---|---|---|---|---|
| OsALMT5 | 224 | 1.07e-14 | GVATCLCTTI | FVMPVWAGEDLH | KLAAGNLDKL |
| AtALMT1 | 182 | 4.43e-14 | GGVSCILISI | FVCPVWAGQDLH | SLLASNFDTL |
| OsALMT9 | 203 | 1.85e-12 | GCAICLFMSL | FVLPNWSGEDLH | SSTVRKFEGL |
| HsGABAa1 | 92 | 8.28e-12 | DHDMEYTIDV | FFRQSWKDERLK | FKGPMTVLRL |
| TaALMT1 | 213 | 1.36e-11 | GVFICLCTTV | FLFPVWAGEDVH | KLASGNLDKL |
| HsGABAb2 | 86 | 9.10e-10 | EVNMDYTLTM | YFQQAWRDKRLS | YNVIPLNLTL |
| GvGLIC | 42 | 4.83e-09 | DKAETFKVNA | FLSLSWKDRRLA | FDPVRSGVRV |
| CeGLC | 115 | 9.34e-09 | VVNMEYSAQL | TLRESWIDKRLS | YGVKGDGQPD |
| DcELIC | 38 | 8.61e-08 | TLEQTYKVDG | YIVAQWTGKPRK | TPGDKPLIVE |

**Supplementary Figure 10 Variation in the consensus motif identified between ALMT and bacterial and animal ion channels.** MEME output[18] for selected ALMT and ion channels used in constructing the human $\alpha_1\beta_2\gamma_2$ $GABA_A$ receptor model[16] which derived the motif of interest [FY][LVF]R[PQ][SV]W[AK][GD][EK][DR]L[HKS]. For full sequences see NCBI/GenBank/EMBL databases under accession numbers HsGABAa1 (NP_507090.2); HsGABAb2 (NP_000804.1); CeGLC1 (NP_507090.1); GvGLIC (568786838); DcELIC (P0C7B7.1).

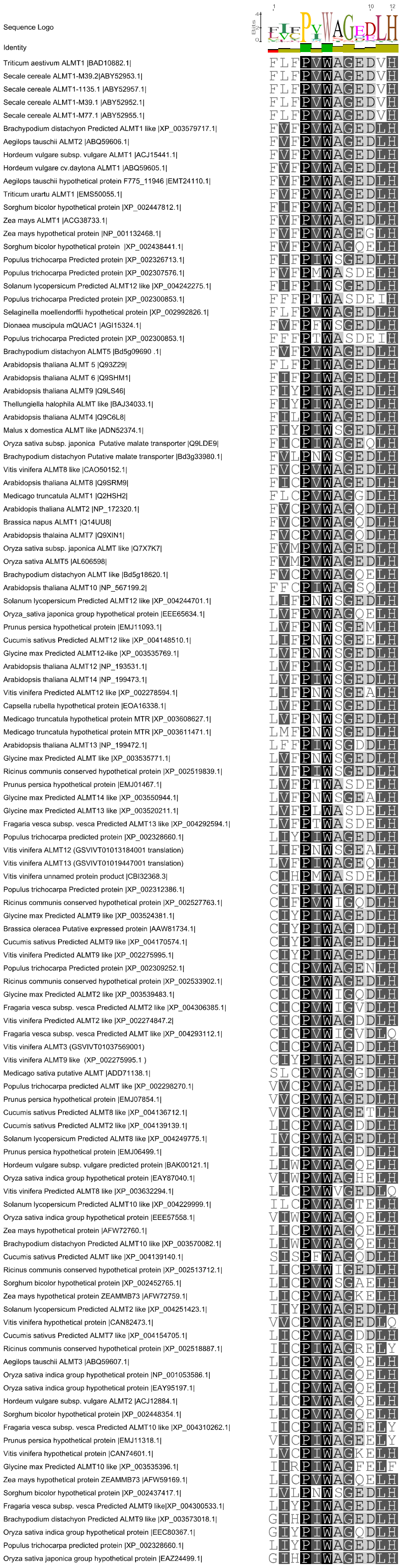


**Supplementary Figure 11 Sequence alignment of 116 ALMT using consensus motif as a BLAST search string.** Highly conserved (100%) residues are shaded in black, 80% similar are shaded grey, while <60% similar are unshaded. Performed using CLUSTAL W2 and Geneious (version 6.1.5).

| Polarity/Charge | | Amino acids | 1 | 2 | 3 | 4 | 5 | 6 | 7 | 8 | 9 | 10 | 11 | 12 |
|---|---|---|---|---|---|---|---|---|---|---|---|---|---|---|
| Hydrophobic | | Alanine [A] | 0 | 0 | 0 | 0 | 0 | 0 | 75.7 | 0 | 0.9 | 2.6 | 0 | 0 |
| Hydrophilic | | Cysteine [C] | 12.9 | 0 | 35.3 | 0 | 0 | 0 | 0 | 0 | 0 | 0 | 0 | 0 |
| Negatively charged | | Aspartic acid [D] | 0 | 0 | 0 | 0 | 0 | 0 | 0 | 0 | 12.2 | 65.2 | 0 | 0 |
| Negatively charged | | Glutamic acid [E] | 0 | 0 | 0 | 0 | 0 | 0 | 0 | 0 | 60.0 | 26.1 | 0 | 0 |
| Hydrophobic | | Phenylalanine [F] | 36.2 | 3.5 | 40.5 | 0 | 1.7 | 0 | 0 | 0 | 0.9 | | 0 | 0.9 |
| Hydrophobic | | Glycine [G] | 2.6 | 0 | 0 | 0 | 0 | 0 | 0 | 93.9 | 1.7 | 0.9 | 0 | 0 |
| Positively charged | | Histidine [H] | 0 | 0 | 2.6 | 0 | 0 | 0 | 0 | 0 | 0.9 | 0 | 0 | 93.9 |
| Hydrophobic | | Isoleucine [Ile] | 5.2 | 51.3 | 0 | 0 | 28.7 | 0 | 4.3 | 0 | 0.0 | 0 | 1.7 | 0 |
| Positively charged | | Lysine [K] | 0 | 0 | 0 | 0 | 0 | 0 | 0 | 0 | 1.7 | 0 | 0 | 0 |
| Hydrophobic | | Leucine [L] | 35.3 | 8.7 | 2.6 | 0 | 0.9 | 0 | 0 | 0 | 0 | 0 | 94.0 | 0 |
| Hydrophobic | | Methionine [M] | 0 | 0.9 | 1.7 | 0 | 1.7 | 0 | 0 | 0 | 0 | 0.9 | 0 | 0 |
| Hydrophilic | | Aspargine [N] | 0 | 0 | 0 | 0 | 10.4 | 0 | 0 | 0 | 0 | 0.9 | 0 | 0 |
| Hydrophobic | | Proline [P] | 0 | 0 | 0 | 100.0 | 0 | 0 | 0 | 0 | 0 | 0 | 0 | 0 |
| Hydrophilic | | Glutamine [Q] | 0 | 0 | 0 | 0 | 0 | 0 | 0 | 0 | 17.4 | 2.6 | 0 | 2.6 |
| Positively charged | | Arginine [R] | 0 | 0 | 0.9 | 0 | 0 | 0 | 0 | 0 | 0.9 | 0 | 0 | 0 |
| Hydrophilic | | Serine [S] | 1.7 | 0 | 0.9 | 0 | 0 | 0 | 19.1 | 6.1 | 0.9 | 0 | 0 | 0 |
| Hydrophilic | | Threonine [T] | 0 | 0 | 0 | 0 | 0 | 0 | 0 | 0 | 0.9 | 0.9 | 0 | 0 |
| Hydrophobic | | Valine [V] | 6.0 | 35.7 | 0 | 0 | 53.0 | 0 | 0 | 0 | 1.7 | 0 | 4.3 | 0 |
| Hydrophobic | | Tryptophan [W] | 0 | 0 | 3.4 | 0 | 0 | 100.0 | 0 | 0 | 0 | 0 | 0 | 0 |
| Hydrophilic | | Tyrosine [Y] | 0 | 0 | 11.2 | 0 | 0 | 0 | 0 | 0 | 0 | 0 | 0 | 2.6 |
| | | Frequently modified residues | F/L | Ile/V | F/C/Y | P | Ile/N | W | A/S | G | E/Q | D/E | L | H |

**Supplementary Figure 12 Variation in the consensus motif identified in ALMT.** Percentage residue frequency extracted from Supplementary Fig. 10. The numbers 1-12 correspond to the amino acid residues 213-224 from TaALMT1. The amino acids most common in these positions across the ALMT are shown in the bottom row of the table. The majority of amino acids in this region are hydrophobic (indicated by red colour).

## Supplementary Table 1 Primers used for cloning and site directed mutagenesis.

| Gene ID | Primer name | Primer sequence | Use |
|---|---|---|---|
| **DQ072260** | TaALMT1_F | 5' ATGGATATTGATCACGGCAGAG 3' | Cloning and Sequencing |
| | TaALMT1_R | 5' TTACAAAATAACCACGTCAGGCAAAGG 3' | |
| | TaMut1_F (F213C) | 5' TGCACCACCGTgTgCCTCTTCCCCG 3' | Mutagenising F213 to C in pGEM-HE |
| | TaMut1_R (F213C) | 5' CGGGGAAGAGGcAcACGGTGGTGCA 3' | |
| | TaMut2_F (F213C,215C) | 5' CACCACCGTgTgCCTCTgCCCCGTCTGGG 3' | Mutagenising F213 and F215 to C in pGEMHE-D |
| | TaMut2_R (F213C,215C) | 5' CCCAGACGGGGcAGAGGcAcACGGTGGTG 3' | |
| | TaMut3_F (F215C) | 5' GTCTTCCTCTgCCCCGTCTGG 3' | Mutagenising F215 to C in pGEMHE-D |
| | TaMut3_R (F215C) | 5' CCAGACGGGGcAGAGGAAGAC 3' | |
| **XM_002275959** | VvALMT9_F | 5' ATGACCGCGAAACTTGGGTCG 3' | Cloning and Sequencing |
| | VvALMT9_R | 5' CTACAACTTCAGCCACCTGCGC 3' | |
| | Vv9Mut1_F (Y237C) | 5' TATGTTTGCTTGTAAATATATGCATCTGTCCCATCT 3' | Mutagenising Y237 to C in pGEMHE-D |
| | Vv9Mut1_R (Y237C) | 5' CAGCCCAGATGGGACAGATGCATATATTTAC 3' | |
| **AT1G08430** | AtALMT1_F | 5' ATGGAGAAAGTGAGAGAGATAGTGAG 3' | Sequencing |
| | AtALMT1_R | 5' TTACTGAAGATGCCCATTACTTAATG 3' | |
| **EF424084** | HvALMT1_F | 5' ATGGAGGTTGATCACCGCATC 3' | Cloning |
| | HvALMT1_R | 5' TCAACTCGCAATGTTGATAGCG 3' | |
| **AT5G46600** | AtALMT13_F | 5' ATGGGGTACAAGGTCGAAGC 3' | Cloning |
| | AtALMT13_R | 5' CTAGATTAACCGGTTCTCAATTCG 3' | |
| **AT5G46610** | AtALMT14_F | 5' ATGTCGGACAGGGTCCATGA 3' | Cloning |
| | AtALMT14_R | 5' CCTAGTGGTT GGCGTGGAGTGA 3' | |
| **Os04g0417000** | OsALMT5_F | 5' GGATCCATGCAATCTGCTGCCGTG 3' | Cloning |
| | OsALMT5_R | 5' CCCGGGTCAACTCATGATGTTGATA 3' | |
| **Os10g0572100** | OsALMT9_F | 5' GGATCCATGGCTTGTGCTCCAGA 3' | Cloning |
| | OsALMT9_R | 5' CCCGGGTTACTCAGCTGCAGTGGAA 3' | |
